# A Novel Lighting OLED Panel Design

**DOI:** 10.3390/molecules21121615

**Published:** 2016-11-25

**Authors:** Enyang Zhang, Weigao Xia, Xiaojian Yan

**Affiliations:** Department of Technical center, Sichuan Changhong Electric Co., Ltd., Chengdu 610041, China; enyang.zhang@changhong.com (E.Z.); weigao.xia@changhong.com (W.X.)

**Keywords:** OLED, photolithography, ITO, insulator, hyper driving

## Abstract

A novel OLED (organic light emitting diode) lighting panel, which uses a special layout design, can reduce the photolithography cycles and process costs and is more reliable. It only needs two steps of photolithography cycles, which include an ITO (InSnO compound transparent oxide) pattern and insulator pattern. There is no need for the metal bus pattern of the ordinary design. The OLED device structure is a type of red–green–blue (RGB)-stacked emitting layer that has a good color index and greater adjustability, which improves the performance of the device. This novel design has the same equipment and material requirement compared to the ordinary design, and it is very beneficial in terms of high volume and low-cost production. It uses a hyper driving method because the entire OLED lighting panel is divided into many sub-emitting units; if one of the sub-emitting units is burned out, it has no effect on the adjacent sub-emitting unit, so the reliability is markedly better than the ordinary design.

## 1. Introduction

The WOLED (white organic light emitting diode) is a new type of illumination device. It is based on OLED technology, which deposits organic emitting materials between the two transparent or metal electrodes. When proper voltage on the anode and cathode electrodes is added, the electrons and holes can be generated and injected into the organic material and recombined in the emitting layer to generate photons out of the device [1].

Using high efficacy material [2], optimum devices [3], tandem structures [4], and outcoupling technology [5], the power efficacy of the WOLED panel can reach 100 lm/W in the market and 400 lm/W in the laboratory [6]. Compared with the LED lighting product, the OLED lighting product is dramatically different in appearance and performance. The OLED lighting panel is large in surface area, low in luminance, thinner than the LED, and usually diffuse in appearance without any optical diffusers [7]. The WOLED has a broad emitting spectrum range and has plenty of different types of materials that can adjust the emitting peak wavelength so as to be a good and green lighting product for human use [8,9]. OLED lighting products are already showing great aesthetic potential as tiles, as soft panels for lighting faces without glare, and as task lights [10].

Many government projects have researched this green lighting technology, for example, the OLLA project in Europe, the DOE project in America, and the NEDO project in Japan [11]. Except for the power efficacy, making a long-lasting, low-cost, and large-area WOLED panel is a major challenge in the OLED lighting industry. 

In order to make a large-area WOLED panel, the present technology uses a metal mesh in the emitting area, which can dissipate the current flow through the entire panel uniformly. As the WOLED panel becomes larger, the metal mesh also becomes longer, and this obviously leads to an IR-drop problem in this type of design. The emitting area, which is far away from the electrode of the panel, is much dimmer than the emitting area near the electrode [12]. People have developed many methods to reduce the non-uniformity of the WOLED panel, such as using a tandem structure that can reduce the resistance of the OLED device and improve the uniformity of the WOLED panel [13], and using a different type of grid design by, say, improving the shape, width, and length of the metal mesh to improve the uniformity and emitting area of the WOLED panel [14].

The improvement of the metal mesh design can achieve good uniformity, but the OLED is a current driving device, and the metal mesh is always resistant, so the metal mesh in the emitting area of the WOLED panel always has non-negligent power dissipation and leads to reliability problems, such as the short between anode and cathode [15], and the thermal problems of the organic materials [16]. 

## 2. Results

We developed a lighting OLED panel that contains nine sub-emitting areas. It only needs two photolithography cycles, and there is no metal mesh inside the emitting area of the entire panel. In order to have a good color index and high efficacy, we made a red and green emitting layer with phosphor material and a blue emitting layer with fluorescent material, and all three emitting layers were stacked together to comprise the OLED device structure.

### 2.1. Lighting OLED Design

#### 2.1.1. Device Structure

**Figure 1:**
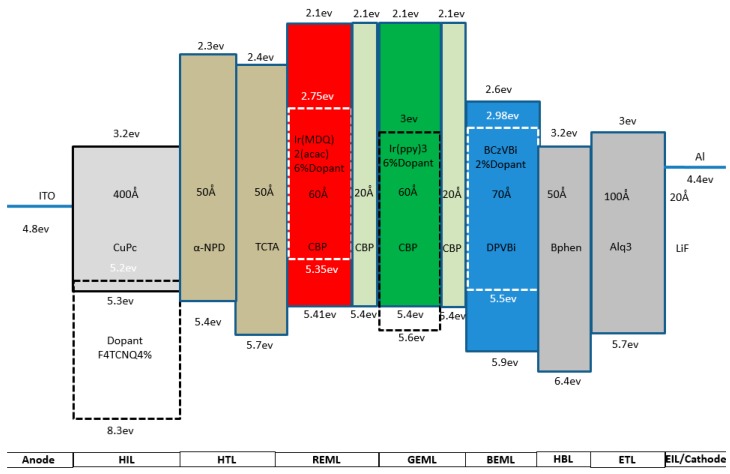
Red–green–blue (RGB)-stacked white organic light emitting diode (WOLED) device structure.

**Figure 2:**
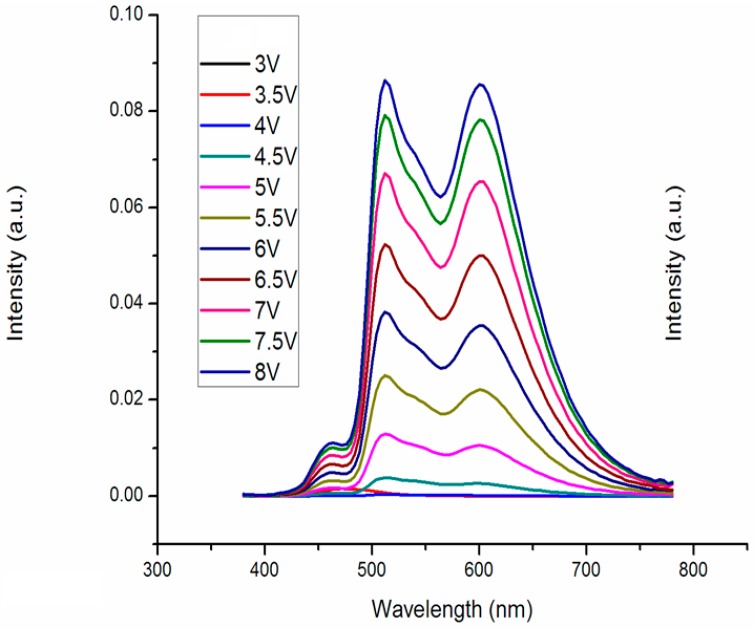
Spectrum of the RGB-stacked WOLED.

In order to have a high CRI (color rendering index) and more adjustability to improve OLED efficacy, we designed a three-peak wavelength WOLED device structure. We used CBP-doped Ir(MDQ)2(acac) as the red emitting layer, CBP-doped Ir(ppy)3 as the green emitting layer, and BCzVBi-doped DPVBi as the blue emitting layer. For the anode and cathode layer, we used ITO and aluminum. We doped HIL (hole injection layer) with F4TCNQ with a 4% ratio to decrease the turn-on voltage, and increase the current density, of the device. For the HTL (hole transfer layer), we used two different HTL materials stacked together, which could generate more holes in the emitting layer and increase the hole transferring ability. We used Bphen as the HBL (hole blocking layer), which can avoid the hole directly transferring to the cathode to decrease the efficacy of the device. For the ETL (electron transfer layer) and EIL (electron injection layer), we used the general material and thickness, which were Alq3@100Å and LiF@20Å. The entire device structure is as follows (Figure 1):

It can be seen from the spectrum diagram in Figure 2 that the red–green–blue (RGB) peak wavelength appeared at 460 nm, 512 nm, 596 nm, which belongs to BCzVBi, Ir(ppy)3, and Ir(MDQ)2(acac).

#### 2.1.2. Lighting Panel Design

Making a large-area lighting WOLED panel is a critical issue. As the light emitting area is increased, there is a non-uniformity of luminance. The electrode of the WOLED panel that is directly connected to the emitting area has the lowest resistance. The current can be easily concentrated in the emitting area and has the highest brightness. However, when the emitting area is far away from the electrode, the current path to it has the largest resistance, which causes a significant voltage drop. The luminance of the emitting area then has the lowest brightness. Even with the metal mesh, which can help to distribute the current flow uniformly, this non-uniformity problem continues to remain. Furthermore, when working for a long time, the WOLED panel can easily burn out due to the hot spot where the current is accumulated and the heat is generated.

**Figure 3:**
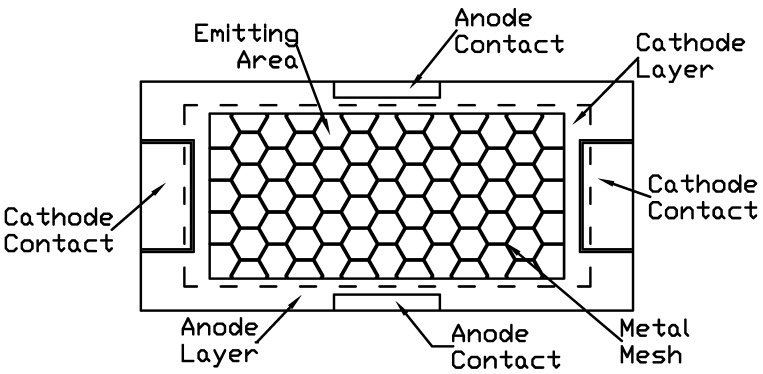
Traditional WOLED layout design.

Figure 3 depicts the general method of the large-area WOLED panel design. In order to have a uniform current flow, there are two pairs of anode/cathode/electrode, and each pair has a vertical alignment between the direction of the anode and cathode. Moreover, in the emitting area, there is a hexagonal metal mesh that can uniformly distribute the current across the emitting area.

Our new design has a very different way of insuring uniform current flow across the emitting area; moreover, it also has a simple photolithography process that can dramatically reduce the total cost of the WOLED panel.

**Figure 4:**
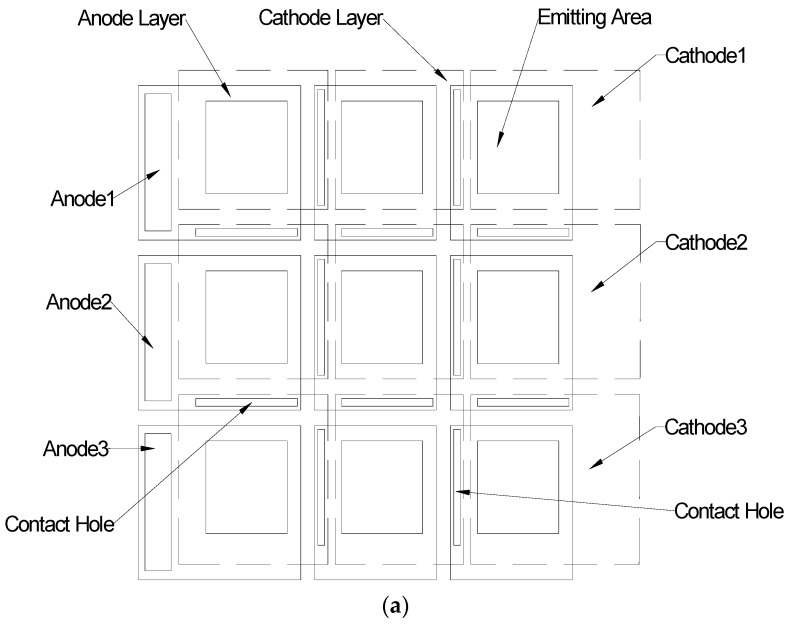

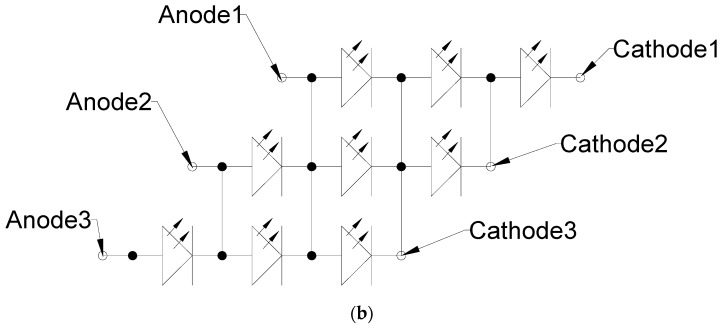
Our novel WOLED layout design and hyper driving method. (**a**) The layout scheme of nine sub-emitting WOLED panel; (**b**) The hyper driving topology of the entire panel.

Figure 4 is our novel WOLED design. There is no metal mesh in the emitting area, so it does not need a metal photolithography cycle in the WOLED manufacture process. The main idea of this design is that we used a hyper driving method for the WOLED panel and then divided the WOLED panel into different sub-emitting areas according to the hyper driving topology. Figure 4a depicts the novel layout design, where there are three pairs of anode/cathode/electrode, and we were able to add more pairs of anode/cathode/electrode. With that, we could divide the entire emitting area of the WOLED panel into nine sub-emitting area aligned lines and rows adjacent to each other, 3 units × 3 units. For every sub-emitting area in the line direction, its anode contacted with the cathode of the prior sub-emitting area; for every sub-emitting area in the row direction, its cathode contacted with the anode of the following sub-emitting area. Every sub-emitting area had a turn-on voltage of 5 V. Figure 4b shows the driving topology for the 3 units × 3 units. hyper driving method. In our test sample, the anode1/anode2/anode3 had voltages of 15 V/20 V/25 V, and the cathode1/cathode2/cathode3 had voltages of 0 V/5 V/10 V.

**Figure 5:**
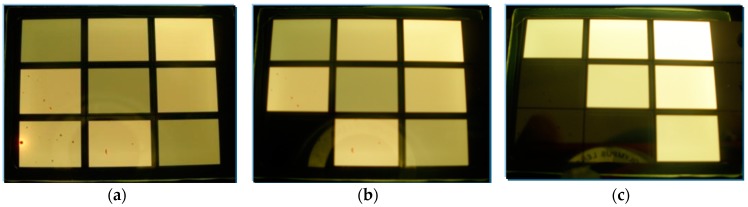
The test sample of our novel design. (**a**) Three pairs of anode/cathode/electrode are all connected; (**b**) anode3 is open; (**c**) anode2 and anode3 are open.

Figure 5 displays the finished test sample of our novel design. Figure 5a is the three pairs of anode/cathode/electrode that are all connected. In Figure 5b, anode3 is open. In Figure 5c, anode2 and anode3 are open. 

## 3. Discussion

Because this novel WOLED panel design does not need a metal mesh in the emitting area, so it only needs two cycles of the photolithography process, which can markedly reduce the material and process cost. Moreover, its reliability is better than the ordinary design, because there is no thermal or short problem induced from the metal mesh and because there are many sub-emitting areas in the panel—if one is burned out, it has no effect on the others. In light of these distinguishing characteristics, this novel lighting OLED design is suitable for OLED lighting products. Our experiment is based on nine sub-emitting areas with a suitable driving method that can be easily extended to larger sizes.

For an OLED device, the reliability is wholly defined at each part of its construction. We constructed a reliability model for the WOLED panel that included a substrate with pattern/chemical materials used in photolithography cycle/organic material/encapsulation cap/driver circuits. All of the units were serially connected as a full reliability model for the WOLED panel. Thus, we used Equation (1) to calculate the reliability of the WOLED panel.

(1) $$T_{F}=\frac{1}{\sum_{i=1}^{5}\lambda s}$$

where T_F_ is the mean time of the failure appearance, and λ_S_ is the failure ratio of each unit.

The failure ratio of each unit was calculated from the failure coefficient of each unit and the desired T_F_. The complexity, maturity, and running environment of each unit resolved the failure coefficient. Based on our observations, we defined the failure coefficient of the five units and the desired T_F_ as 10,000 h, and we multiplied the failure coefficient by the 90% reciprocal of the T_F_, yielding the failure ratio of each unit as shown in Table 1.

Compared with our novel design, the ordinary WOLED panel design needs a metal grid in the emitting area, so the complexity of the substrate is higher. Moreover, because the ordinary design needs one more photolithography cycle in the process, the effect of the chemical material is also higher. Both of these lead to a high failure coefficient and high failure ratio of the reliability units and the entire device. Hence, the ordinary WOLED panel design has worse reliability compared with our novel design.

## 4. Materials and Methods 

We made the test sample in our 200 mm × 200 mm OLED research line: We used a 0.7 mm thick soda-lime glass substrate with a size of 200 mm × 200 mm, which was coated with SiO_2_ (1500 Å)/ITO (1500 Å) sequentially. The substrate was firstly cleaned in 18 MΩ DI water with bubble and rinse cycles, and we used a soft brush to clean the ITO surface of the substrate dipped in weak-alkaline cleaner. After the brushing process, the substrate was put into an ultrasonic tank filled with weak-alkaline cleaner for about 20 min, and the spin-dry method was used to desiccate the substrate. After finishing the cleaning process, the substrate was sent into the track machine, coated with positive photo-resist, and baked at 130 °C for about 90 s. After the coating process, the substrate was sent into the exposure machine worked in i-line to be exposed with a dose of about 16 mj. Again, the substrate was sent into the track machine, where the exposed substrate was developed in a 2.37% TMAH (tetramethylammonium hydroxide) developer and baked at 130 °C for about 120 s. After the developing process, we put the substrate with a baked photo-resist pattern into a wet station, where we used a HCL and HNO_3_ mixture as the etchant to etch the ITO material, which was not covered by baked photo-resist. After the etching process, we used an organic stripper to peel off the baked photo-resist at 70 °C for about 20 min. After the stripping process, we cleaned the substrate with a well-done ITO pattern in order to prepare for the next photolithography cycle. 

In the insulator photolithography process, there is no cleaning, etching, or stripping process; instead, the photolithography process needs a curing process in a curing oven worked at about 250 °C in order to harden the photo resin and create a greater adhesive force to the substrate.

After the 2 cycles, the photolithography process was finished, and the sub-emitting area pattern was finished via ITO and an insulator pattern. We put the substrate with a sub-emitting area pattern into an O_2_ plasma chamber to clean the organic particles on the substrate at 70 W of power for about 90 s. Then, the substrate with a sub-emitting area pattern was sequentially deposited with a HIL/HTL/REML (red emitting layer)/GEML (green emitting layer)/BEML (blue emitting layer)/HBL/ETL/EIL and a cathode layer with a cluster type evaporation system. The cluster type evaporation system has a low-temperature heating source for organic material and an e-beam source for inorganic material. All deposition chambers maintained their vacuum level at 10^−7^ torr.

After the organic material deposition process, we used a glass cap cleaned via the UV method for 20 s and stuck with a gas getter to encapsulate the WOLED panel in the glove box filled with pure N_2_ to insure the organic material on the substrate was free of erosion by O_2_ and H_2_O. The encapsulation pressure was maintained at 1 ATM, and the temperature was aimed at −70 °C, which is the dew temperature of H_2_O. In order to isolate the organic material from the atmosphere, we dispensed UV resin around the WOLED panel and exposed it to 365 nm UV light for 90 ms to cure the resin.

## 5. Conclusions

We developed a novel lighting WOLED panel that uses a RGB-stacked WOLED device structure, and the test results of this WOLED device are shown in Table 2.

Our novel design does not need a metal mesh in the emitting area, so there is no concern for a heat accumulation problem which lead to the WOLED panel burned out. Moreover, this novel design can reduce the material and process cost of WOLED panels because there is no need for metal deposition or photolithography processes. Moreover, with the hyper driving method used in our test sample, we can easily adjust the voltage of the three pairs of electrodes (15 V/20 V/25 V; 0 V/5 V/10 V) to ensure the uniformity of the entire panel. When a sub-emitting area is burned out, the adjacent area will continue to emit light.

## Figures and Tables

**Table 1:** Failure coefficient for each unit of the WOLED panel.

Unit Name	Failure Coefficient	Failure Ratio
Substrate	0.226633	2.0397 × 10^−5^ /h
Chemical Material	0.164607	1.48146 × 10^−5^ /h
Organic Material	0.255499	2.29949 × 10^−5^ /h
Encapsulation Cap	0.139129	1.25216 × 10^−5^ /h
Driver Circuit	0.214132	1.92719 × 10^−5^ /h

**Table 2:** Test results of test sample.

Working Voltage	Current Density	Current Efficiency	Power Efficiency	Brightness
4 V	0.1 mA/cm^2^	14 cd/A	11 lm/W	15 cd/m^2^
4.5 V	0.7 mA/cm^2^	27 cd/A	19 lm/W	193 cd/m^2^
5 V	2.5 mA/cm^2^	28 cd/A	18 lm/W	692 cd/m^2^
